# Functional characterization of a cystatin from the tick *Rhipicephalus haemaphysaloides*

**DOI:** 10.1186/s13071-015-0725-5

**Published:** 2015-03-03

**Authors:** Yujian Wang, Yongzhi Zhou, Haiyan Gong, Jie Cao, Houshuang Zhang, Xiangrui Li, Jinlin Zhou

**Affiliations:** Key Laboratory of Animal Parasitology of Ministry of Agriculture, Shanghai Veterinary Research Institute, Chinese Academy of Agricultural Sciences, Shanghai, 200241 China; College of Veterinary Medicine, Nanjing Agricultural University, Nanjing, 210095 China; Jiangsu Co-innovation Center for Prevention and Control of Important Animal Infectious Diseases and Zoonoses, Yangzhou, 225009 China

**Keywords:** *Rhipicephalus haemaphysaloides*, Cystatin, Inhibitory activity, Embryonic development

## Abstract

**Background:**

Ticks and tick-borne diseases affect animal and human health worldwide and cause significant economic losses in the animal industry. Functional molecular research is important to understand the biological characteristics of ticks at the molecular level. Enzymes and enzyme inhibitory molecules play very important roles in tick physiology, and the cystatins are tight-binding inhibitors of papain-like cysteine proteases. To this end, a novel cystatin, designated RHcyst-1, was isolated from the tick *Rhipicephalus haemaphysaloides.*

**Methods:**

The full-length gene of RHcyst-1 was cloning by RACE. The recombinant protein of RHcyst-1 was expressed in a glutathione S-transferase (GST)-fused soluble form in *Escherichia coli*, and its inhibitory activity against cathepsin L, B, C, H, and S, as well as papain, was identified by fluorogenic substrate analysis. Expression analysis of RHcyst-1 at different tick stages was performed by quantitative reverse transcription - PCR (qRT-PCR). An RNAi experiment for RHcyst-1 was performed to determine its function for tick physiology.

**Results:**

The full-length cDNA of RHcyst-1 is 471 bp, including an intact open reading frame encoding an expected protein of 98 amino acids, without a signal peptide, having a predicted molecular weight of ~11 kDa and an isoelectric point of 5.66. A sequence analysis showed that it has significant homology with the known type 1 cystatins. The results of proteinase inhibition assays showed that rRHcyst-1 can effectively inhibit the six cysteine proteases’ enzyme activities. An investigation of the RHcyst-1 genes’ expression profile showed that it was more richly transcribed in the embryo (egg) stage. A disruption of the RHcyst-1 gene showed a significant decrease in the rate of tick hatching.

**Conclusions:**

Our results suggested that RHcyst-1 may be involved in the early embryonic development of ticks.

## Background

Ticks rank first as arthropod vectors of fungi, protozoa, rickettsiae, bacteria, and viruses, which cause diseases in non-human vertebrates, and rank second only to mosquitoes as vectors of human pathogens [1]. Cystatins are classified, based on characteristic sequence motifs and the number of conserved cystatin domains, into four subfamilies: the type 1 cystatins (also known as stefins), the type 2 cystatins, the type 3 cystatins (kininogens), and the type 4 cystatin-like proteins (fetuins and histidine-rich proteins) [2]. Type 1 cystatins are cytoplasmic proteins that do not have signal peptides; however, the type 2 cystatins are secretion-type proteins containing signal peptides. Cystatins are present in a wide range of organisms, such as vertebrates, invertebrates, and plants, as well as protozoa [3,4]. They are involved in various vertebrate biological processes, such as antigen presentation, immune system development, epidermal homeostasis, neutrophil chemotaxis during inflammation, and apoptosis [5-8]. Over the last decade, several cystatins from different hard and soft ticks were identified and biochemically analyzed to determine their roles in the physiology and blood-feeding lifestyle of ticks [9].

*Rhipicephalus haemaphysaloides* is a widespread tick species in China and other south Asian countries, where it transmits animal babesiosis and human Kyasanur Forest disease [10]. In this paper, we report on a novel cystatin molecule, named RHcyst-1, identified from *R. haemaphysaloides*. This molecule exhibited significant inhibitory activities against cysteine proteinases and may be involved in tick embryonic development.

## Methods

### Ticks and animals

The Hubei strain of *R. haemaphysaloides* has been maintained by feeding on rabbits for several generations in our laboratory at the Chinese Academy of Agricultural Sciences (Shanghai, China) [10]. The experimental animals were treated following the approved guidelines from the Animal Care and Use Committee of the Shanghai Veterinary Research Institute (SOP-1104-003).

### Cloning the full-length gene by RACE and bioinformatic analysis

Rapid amplification of cDNA ends (RACE) was conducted using a SMARTer RACE cDNA amplification kit (Clontech, San Jose, CA, USA) following the manual’s instructions. Cloning was performed using primers from highly conserved regions of cystatin [2]. The gene-specific primers used were 5′-AAGGATGCCGATGACACAGTC-3′ and 5′-CCCTGGAAAGCCTTGTGCGC-3′. The cDNA template primed by an adapter-linked oligodT primer (Clontech) was synthesized from 5 μg of total RNA extracted from ticks that had been partially fed for 4 days. After two rounds of PCR, the PCR fragments were cloned into the pGEM-T plasmid (Promega, Madison, WI, USA) and sequenced. A BLASTx homology search revealed a cDNA encoding a cystatin-like polypeptide. Following contig assembly and singleton identification, gene-specific PCR primers were designed and used to clone the full-length cDNA [11].

### Expression and purification of rRHcyst-1 in *Escherichia coli*

The open reading frame (ORF) of the cystatin gene in the pGEM-T vector was subcloned into the pGEX-4T-1 *E. coli* expression vector (Amersham Pharmacia Biotech, Piscataway, NJ, USA). The accuracy of the insertion in the resulting plasmid was confirmed by sequencing. The cystatin gene was expressed as a glutathione S-transferase (GST)-fusion protein in the *E. coli* BL21 (DE3) strain according to the manufacturer’s instructions (Amersham Pharmacia Biotech). The resulting *E. coli* cells were washed three times with phosphate-buffered saline (PBS), lysed in PBS containing 1% Triton X-100, sonicated, and then centrifuged at 12,000 × *g* for 10 min at 4°C. Supernatants containing the soluble GST fusion protein were purified with glutathione-Sepharose 4B beads (Amersham Pharmacia Biotech) according to the manufacturer’s instructions. The purified proteins were dialyzed against PBS for further use. The empty pGEX-4T-1 vector was used to produce the control GST protein, which was expressed and purified using the same procedure as that for the cystatin-GST fusion protein. Recombinant protein expression and purification analyses were carried out by standard SDS**–**PAGE [12].

### Proteinase inhibition assays

To calculate the inhibitory activity of the recombinant protein, the concentration of rRHcyst-1 at which a 50% inhibition of the proteolytic enzymes’ activities was achieved (IC_50_) was measured. Recombinant protein was preincubated with each enzyme (0.15 μM) in an assay buffer for 30 min. Then, 0.25 mM of the protease-specific substrates was added to each well and residual enzyme activity monitored [13]. The GST protein was used as control. Enzymes used were as follows: cathepsin L, C, B, S and H, as well as papain. All of these enzymes were purchased from Sigma Company (St. Louis, USA). The assay buffer used consisted of 100 mM sodium acetate, pH 5.5, 100 mM NaCl, 1 mM EDTA, 1 mg/ml cysteine, and 0.005% TritonX-100. The substrates purchased (Sigma company) were as follows: Z-Phe-Arg-AMC·HCl for papain, cathepsin L and cathepsin B; Pro-Arg-4-methoxy-β-naphthylamide acetate salt for cathepsin C; Arg-NMec·HCl for cathepsin H; and Ac-Lys-Gln-Lys-Leu-Arg-AMC for cathepsin S.

### Expression analysis of RHcyst-1 in ticks at different developmental stages by qRT-PCR

Relative quantification was carried out using 100 ng of cDNA prepared from the eggs, larvae, nymph, and adult cDNA. To normalize the obtained gene expression, the tick elongation factor 1-alpha gene was selected as a housekeeping gene, as described previously [14]. The specific primers used to quantify the cystatin and elongation factor 1-alpha were 5′-CACAGTCAGGGAGATTTGCG-3′ and 5′-TGCGTGCGATACTTCAGAGG-3′ for RHcyst-1, and 5′-CGTCTACAAGATTGGTGGCATT-3′ and 5′-CTCAGTGGTCAGGTTGGCAG-3′ for elongation factor 1-alpha. The amount of mRNA transcripts of the target genes present in the adult samples were considered equivalent to 1 and were used as references for the expression levels in the other stages. Cycling parameters for all amplifications were 5 min at 95°C followed by 30 cycles of 15 s at 95 C, 30 s annealing at 60°C, and extension at 72°C for 30 s. qPCR was performed using Platinum SYBR Green qPCR SuperMix kit (Invitrogen) using an Applied Biosystems 7500 thermocycler (Applied Biosystems Inc, Foster City, CA, USA). To ensure primer fidelity, dissociation curve analyses and gel electrophoresis of target gene amplicons were performed for each sample following the qPCR step. All qPCR amplifications were performed in triplicate and repeated twice, with the mean values considered for comparison. To check for genomic DNA contamination, controls lacking reverse transcription were performed.

### RNA interference of RHcyst-1

cDNA of RHcyst-1 in the pGEM-T vector was amplified by PCR using oligonucleotides, including the T7 promoter sequence at the 5′ end of both primers, as follows: 5′-GGATCCTAATACGACTCACTATAGGGTAGCAGCGCGCGCGATTAG-3′ and 5′-GGATCCTAATACGACTCACTATAGGCGCGTAAAATTTGTTCCTT-3′ for the gene encoding RHcyst-1, and 5′-GGATCCTAATACGACTCACTATAGGGCTTCCATCTTCCAGGGATACG-3′ and 5′-GGATCCTAATACGACTCACTATAGGCGTCCACAAACACAACTCCTCC-3′ for the gene encoding luciferase. The PCR products were gel-purified and used to synthesize RNA by in vitro transcription with T7 RNA polymerase (T7 RiboMAX™ Express RNAi System, Promega, Madison, WI, USA) according to the manufacturer’s protocol. The double-stranded (ds) RNA injection was administered as described previously [15]. In total, 1 μg of the RHcyst-1 dsRNA in 0.5 μl of an injection buffer (10 mM Tris and 1 mM EDTA, pH 7.4) was microinjected into unfed female ticks from the same batch of engorged nymphal ticks (105 ticks in each group). Twelve unfed female ticks that had been removed from the host 5 days after feeding for real-time PCR analysis were not included. In the control group, another 105 unfed ticks were injected with luciferase dsRNA constructed from the vector DNA of pGEM-luc (Promega). The ticks were allowed to rest for 1 day at 25°C and 95% relative humidity, and then each group of ticks (RHcyst-1 and control) was allowed to feed on three rabbits (35 ticks on each rabbit). The attachment rate 24 h after putting the adult ticks onto the host ears, the engorgement rate, the death rate and the larval hatching rate were measured to evaluate the RHcyst-1 functions in tick physiology. To confirm gene-specific silencing, real-time quantitative PCR using SYBR Green technology and gene-specific primers was performed as above described. Statistical analyses for significant differences in the results of the RNAi test were calculated using Student’s *t* test (P < 0.05).

## Results

### Cloning and sequence analysis of the full-length cDNA encoding *R. haemaphysaloides* cystatin

**Figure 1:**
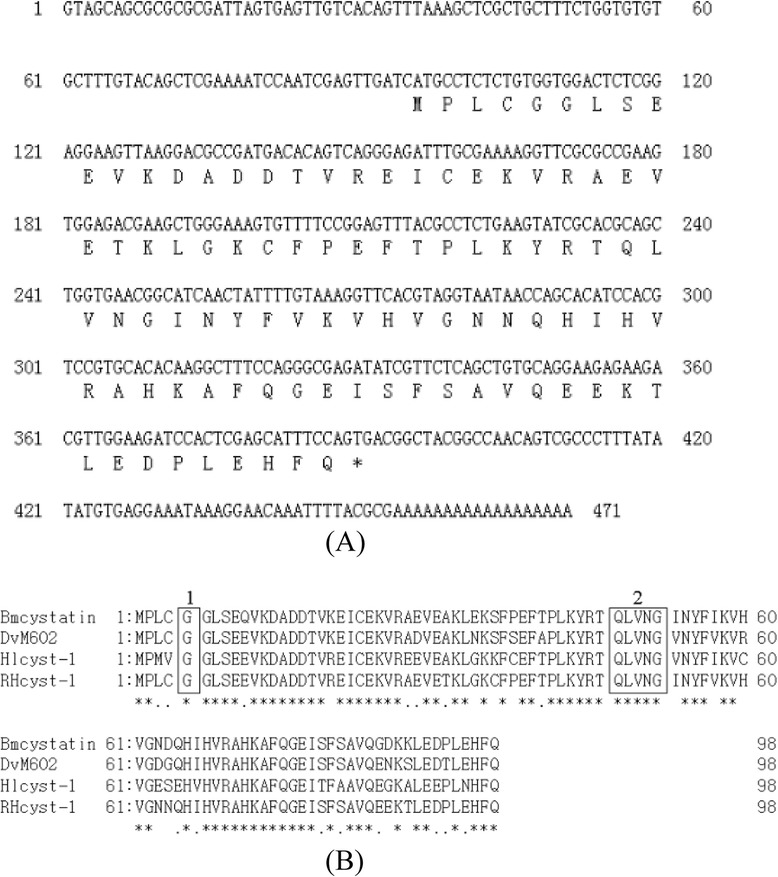
**Analysis of the structure of a novel type 1 cystatin, RHcyst**-**1,** **isolated from the tick** ***Rhipicephalus haemaphysaloides*** **. (A)** cDNA and putative amino acid sequences. **(B)** Putative amino acid alignment of RHcyst-1 with other tick type 1 cystatins: Bmcystatin of *Boophilus microplus* (GenBank accession number: ABG36931); DvM602 of *Dermacentor variabilis* (GenBank accession number: ACF35512); and Hlcyst-1 of *Haemaphysalis longicornis* (GenBank accession number: ABZ89553). The conserved cystatin active sites are boxed (1: N-terminal conserved glycine; 2: QXVXG conserved motif).

We found a novel cystatin gene, RHcyst-1, from the tick *R. haemaphysaloides* using the RACE method. The full-length cDNA of RHcyst-1 is 471 bp, as shown in Figure 1A, including an intact ORF encoding an expected protein of 98 amino acids, with a predicted protein molecular weight of ~11 kDa and an isoelectric point of 5.66. A sequence analysis demonstrated that RHcyst-1 possesses a conserved N-terminal glycine and the QXVXG motif, which is highly conserved in various type 1 cystatins. SMART analysis (http://smart.embl-heidelberg.de/) detected the cystatin**-**like domain in the putative amino acid sequence (position 3–97). A BLASTP analysis of the predicted polypeptide sequence against all non-redundant databases accessed through GenBank revealed significant similarity scores with members of the cystatin type 1 family of other species. The identities of the putative RHcyst-1 amino acid sequences with the type 1 cystatin of *Boophilus microplus* (GenBank accession number: ABG36931), *Dermacentor variabilis* (GenBank accession number: ACF35512), and *Haemaphysalis longicornis* (GenBank accession number: ABZ89553) were 90%, 86%, and 81%, respectively, as shown in Figure 1B. The amino acid analysis using the Signal P program did not reveal the presence of a signal peptide. The sequence of the gene encoding RHcyst-1 have been submitted to GenBank under the accession number KM588364.

### Expression and purification of rRHcyst-1

**Figure 2:**
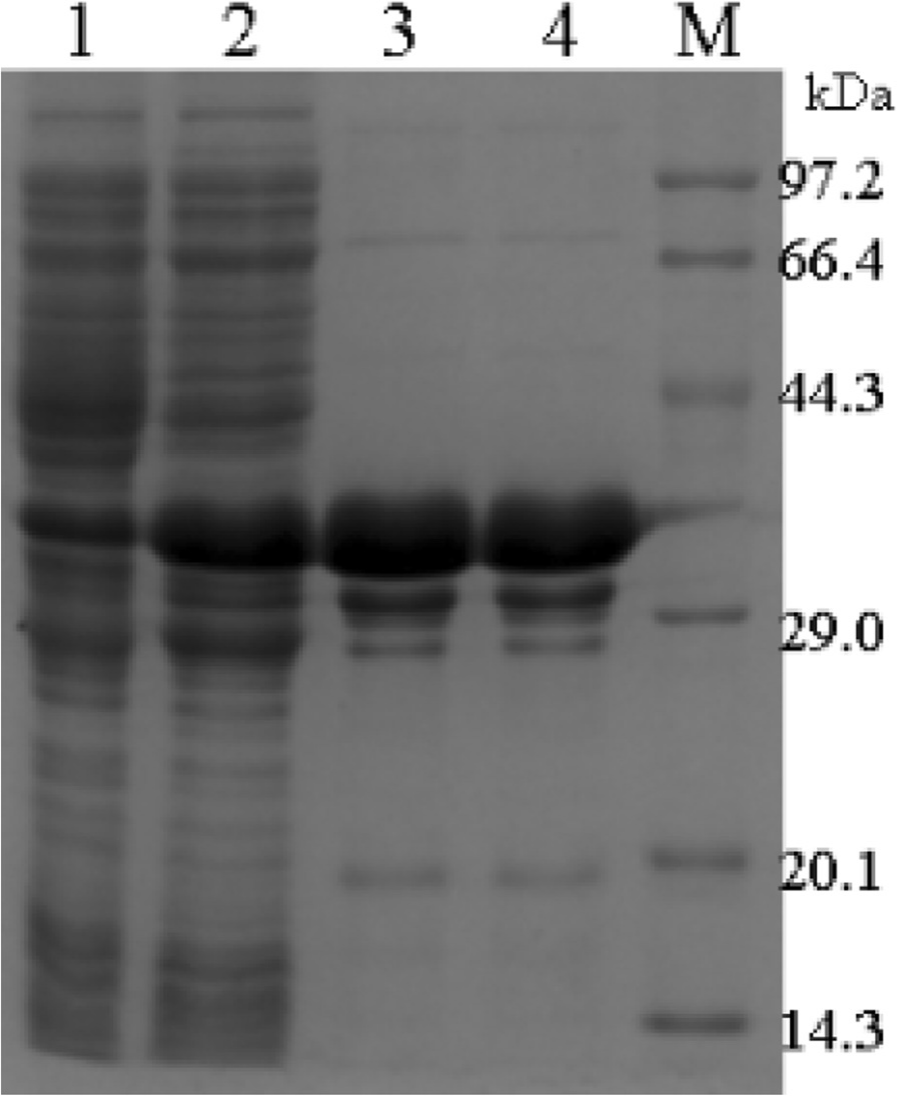
**Expression and purification of the recombinant protein of RHcyst-** **1,** **a novel type 1 cystatin isolated from the tick** ***Rhipicephalus haemaphysaloides,*** **in a GST-** **fused soluble form,** **rRHcyst-** **1-** **GST,** **in** ***Escherichia coli*** **.** A total of ~10 μg recombinant protein or bacterial lysate was electrophoresed in a 12% SDS-polyacrylamide gel, and then stained with Coomassie blue. Lane 1: The supernatant of uninduced cell lysate; lane 2: The supernatant of induced cell lysate; and lanes 3 and 4: purified RHcyst-1 recombinant protein.

The gene encoding RHcyst-1 was ligated into the bacterial expression vector pGEX-4T-1, and the recombinant was successfully expressed as GST-fusion protein with an expected size of 37 kDa (Figure 2). rRHcyst-1 was expressed in a soluble form and then purified by affinity chromatography using Sepharose 4B columns according to the manufacturer’s instructions. rRHcyst-1 was more than 95% pure as estimated by SDS**-**PAGE analysis.

### Proteinase inhibition assays

**Figure 3:**
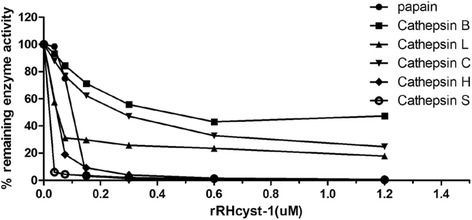
**Inhibition of protease activities by the recombinant protein of RHcyst-** **1,** **a novel type 1 cystatin isolated from the tick** ***Rhipicephalus haemaphysaloides*** **.** Cathepsin L, B, C, H, and S and papain were incubated with each of the substrates in the presence of different concentrations of rRHcyst-1. Incubation of cathepsins without rRHcyst-1 resulted in 100% enzyme activity.

To investigate the efficiency of RHcyst-1 in inhibiting its overlapping target enzymes, purified recombinant cystatin was used to test the inhibitory activity against cysteine proteases. The results show that: rRHcyst-1 can effectively inhibit cathepsin L, B, C, H, and S, as well as papain, enzyme activities, as shown in Figure 3 and Table 1. rRHcyst-1 effectively inhibited cathepsin S, whereas it showed relatively less inhibitory activity against cathepsin B.

**Table 1:** **Protease inhibition assays**

**Enzyme**	**Enzyme concentration**	**RHcyst**-**1 IC**_**50**_ **and 95%** **confidence intervals**
Papain	150 nM	89 nM (86 to 92.1 nM)
Cathepsin B	150 nM	594.5 nM (305.5 to 1157 nM)
Cathepsin L	150 nM	34 nM (9.2 to 125.5 nM)
Cathepsin C	150 nM	281.8 nM (244.5 to 324.7 nM)
Cathepsin H	150 nM	23.7 nM (22.2 to 25.4 nM)
Cathepsin S	150 nM	0.3 nM (~ to 1.4 nM)

The concentration of a novel type 1 cystatin, RHcyst-1, isolated from the tick *Rhipicephalus haemaphysaloides*, at which 50% of the proteolytic enzymes’ activity is inhibited (IC_50_).

### Expression analysis of RHcyst-1 in ticks at different developmental stages by qRT-PCR

**Figure 4:**
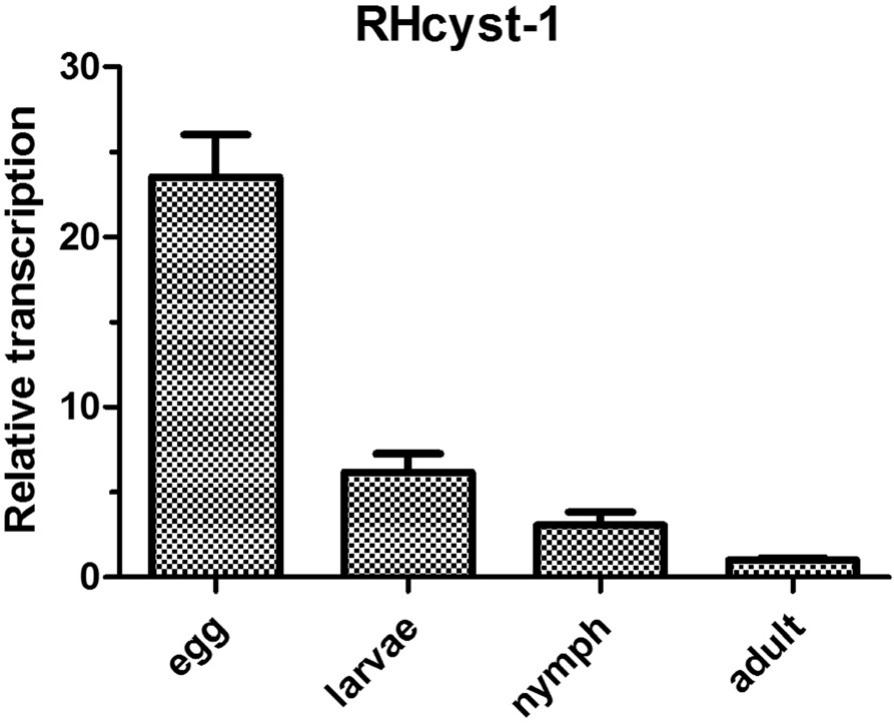
**Gene expression patterns of a novel type 1 cystatin,** **RHcyst-** **1,** **isolated from the tick** ***Rhipicephalus haemaphysaloides*** **in different developmental stages.** The relative expression (%) refers to adult stage RHcyst-1 expression (1 unit). Each column represents the mean and standard deviation (error bars) of three analyses.

To determine the expression profile of RHcyst-1, total RNA samples from four different tick developmental stages were subjected to real-time PCR. RHcyst-1 mRNA transcripts were detected in eggs (embryos), larvae, nymphs, and adults. RHcyst-1 was most highly expressed in the embryo stage, as shown in Figure 4.

### RNA interference of RHcyst-1

**Figure 5:**
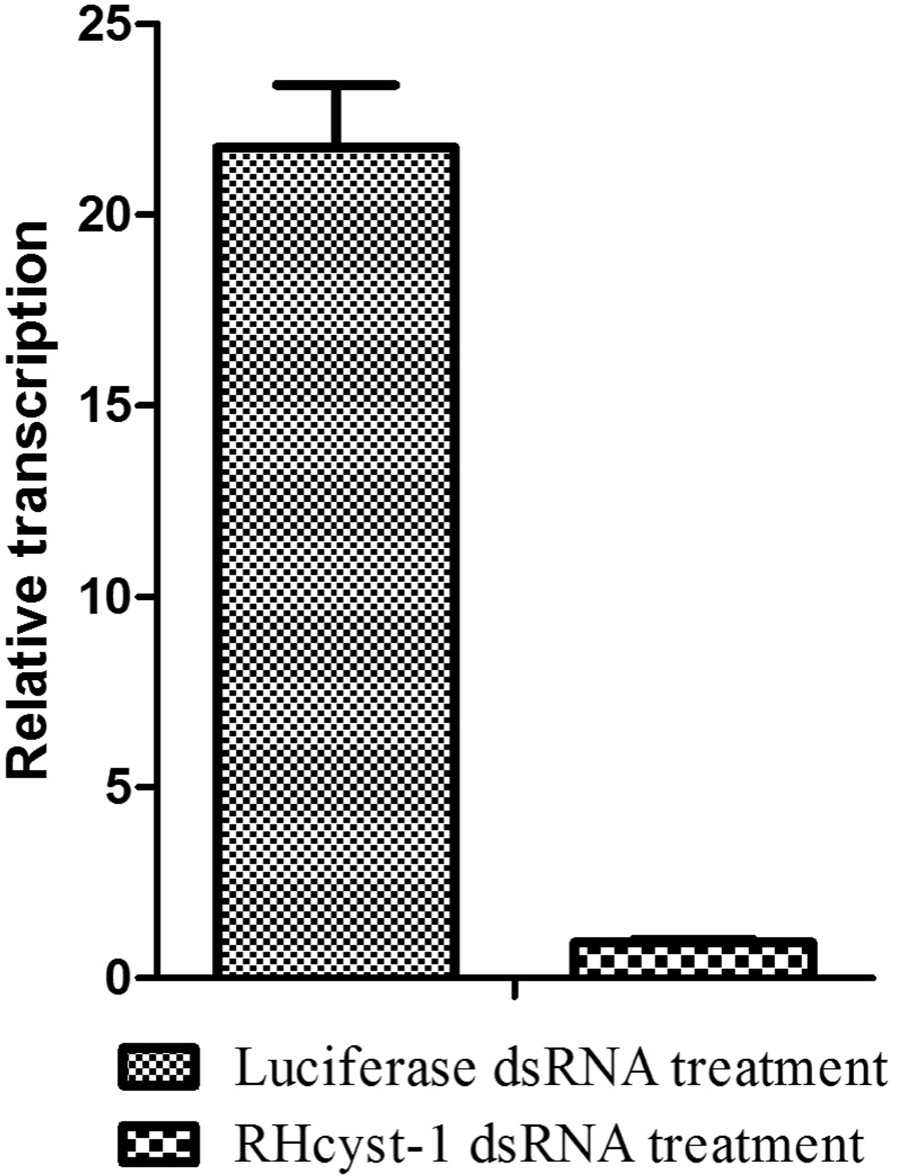
**Expression of a novel type 1 cystatin,** **RHcyst-** **1,** **isolated from the tick** ***Rhipicephalus*** **haemaphysaloides,** **dsRNA or luciferase dsRNA treatments in female ticks.** The relative expression (%) refers to the RHcyst-1 expression (1 unit) in RHcyst-1 dsRNA treated female ticks. Each column represents the mean and standard deviation (error bars) of three analyses.

Unfed adult ticks were injected with RHcyst-1 or luciferase dsRNA and then allowed to feed on a rabbit. Gene-specific silencing was confirmed by real-time PCR. Within 5 days of dsRNA treatment, 95.6% of the RHcyst-1 transcripts in ticks were silenced as shown in Figure 5. The attachment rate 24 h after putting adult ticks onto the host ears, the engorgement rate, the death rate, and the larval hatching rate were measured to evaluate the RHcyst-1 function in tick physiology. Compared with luciferase-injected ticks, there was a significant effect on the attachment and hatching rates, but no significant effect on engorgement or death rates. Table 2 shows that the attachment and hatching rates in the RHcyst-1 dsRNA group were 48.6% and 69.1%, respectively, whereas those in the control group were 63.8% and 80.4%, respectively.

**Table 2:** **Effect of a novel type 1 cystatin, RHcyst-1, isolated from the tick** ***Rhipicephalus haema physaloides*** **, dsRNA treatment on ticks**

**Influencing parameter**	**Control**	**RHcyst-1**	**Significant difference**	**P-value**
Attachment rate after 24 h (%)	63.8 ± 3.3	48.6 ± 2.9	Yes	0.0038
Engorgement rate (%)	42.9 ± 5.7	40 ± 2.9	No	0.4818
Death rate (%)	56.2 ± 1.6	59.0 ± 1.6	No	0.1012
Hatching rate (%)	80.4 ± 4.1	69.1 ± 2.4	Yes	0.0146

Only female ticks were tested; values were expressed as an average±standard error. Significant differences were calculated using Student’s *t* test.

## Discussion

The present study describes the sequence of a novel cystatin, RHcyst-1, from the tick *R. haemaphysaloides*. The characteristics of the putative RHcyst-1 amino acid sequence indicate that it is a member of the type 1 cystatins.

The capacities of various cystatins to inhibit the activity of cysteine proteases have been characterized. In this study, the GST-fused recombinant cystatin efficiently inhibited the activity of cathepsin L, B, C, H, and S, and papain. rRHcyst-1 effectively inhibited cathepsin S, and it also had quite a high inhibitory efficiency against cathepsin L. However, rRHcyst-1 showed relatively less inhibitory activity against cathepsin B, perhaps due to an occluding loop in the cathepsin B active site that limits the access of both substrates and inhibitors to the active site [16]. The lack of inhibitory activity has been documented previously for other cystatins [12,13,17-19]. In recent studies, cathepsin L and S were considered to be effectively controlled targets of tumor cells, because they play key roles in the invasion and migration of tumor cells [20,21]. The SV-cystatin from snake venom was found to inhibit the invasion and metastasis of mouse melanoma cells and human gastric carcinoma cells [22]. Thus, based on this information, RHcyst-1 may be an important candidate molecule for anti-cancer drug research and development in the future.

All known type 1 tick cystatins, except RHcyst-1, were discovered from five different hard tick species of four genera. Only two type 1 tick cystatins have been biochemically analyzed thoroughly enough to identify their roles in tick physiology [9]. Bmcystatin (GenBank accession number: ABG36931) has a 70% sequence identity with a cytoplasmic salivary protein of *Ixodes scapularis* (GenBank accession number: AAY66864), which was the first type 1 cystatin identified in the *I. scapularis*’ sialome [18,23]. Lima et al. [18] suggested a role for Bmcystatin in the embryogenesis of *R. microplus* because this cystatin was found to inhibit, in addition to human cathepsin L, a vitellin-degrading cysteine endopeptidase of *R. microplus*. Hlcyst-1 (GenBank accession number: ABZ89553) was localized in epithelial cells of the tick midgut alongside with HlCPL-A, a cathepsin L-like cysteine protease of *H. longicornis* [24]. HlCPL-A may play a role in the digestion of host hemoglobin in ticks since it is able to degrade bovine hemoglobin [25]. Hlcyst-1 efficiently inhibited the hemoglobinolytic activity of HlCPL-A, and both gene transcripts were upregulated during the blood feeding of *H. longicornis*, with their strongest expression levels occurring at 48 h in the midgut cells [24]. Thus, Hlcyst-1 seems to function as a regulator for digesting blood in ticks.

## Conclusions

In this study, RHcyst-1 was found to be highly expressed in the embryo (egg) stage, and we hypothesize that it plays a role in early embryonic development. To further confirm the function of RHcyst-1 in tick physiology, an RNAi experiment was performed. The disruption of the RHcyst-1 gene led to a significant decrease in the rate of egg hatching. However, there are several cystatin homologs which may compensate the role of the silenced cystatin, thus affect the result of RHcyst-1 RNAi. And this hypothesis will be tested in our future work. In conclusion, our results suggested that RHcyst-1 may be involved in tick early embryonic development.
